# Three lessons on diabetes for global health professionals, researchers and policy-makers from the people of Ga Mashie

**DOI:** 10.3389/fnut.2025.1534450

**Published:** 2025-03-14

**Authors:** Olutobi Adekunle Sanuade, Lydia O. Okoibhole, Ernestina K. Dankyi, Daniel Strachan, Leonard Baatiema, Sandra Boatemaa Kushitor, Raphael B. Awuah, Mawuli K. Kushitor, Samuel Amon, Irene Akwo Kretchy, Daniel Arhinful, Edward Fottrell, Megan Vaughan

**Affiliations:** ^1^Department of Population Health Sciences, Division of Health System Innovation and Research, Spencer Fox Eccles School of Medicine at the University of Utah, Salt Lake City, UT, United States; ^2^Institute for Global Health, University College London, London, United Kingdom; ^3^Centre for Social Policy Studies, University of Ghana, Accra, Ghana; ^4^Nossal Institute for Global Health, University of Melbourne, Melbourne, VIC, Australia; ^5^Department of Health Policy, Planning and Management, School of Public Health, University of Ghana, Accra, Ghana; ^6^Department of Community Health, Ensign Global College, Kpong, Ghana; ^7^Regional Institute for Population Studies, University of Ghana, Accra, Ghana; ^8^Vital Strategies, New York, NY, United States; ^9^Department of Health Policy Planning and Management, School of Public Health, University of Health and Allied Sciences, Ho, Ghana; ^10^Department of Pharmacy Practice and Clinical Pharmacy, School of Pharmacy, University of Ghana, Accra, Ghana; ^11^Noguchi Memorial Institute for Medical Research, University of Ghana, Accra, Ghana; ^12^Institute of Advanced Studies, University College London, London, United Kingdom

**Keywords:** diabetes, cultural context, Ga Mashie, Ghana, global health

## Abstract

**Background:**

Diabetes is a leading cause of death globally, with significant burdens in low- and middle-income countries (LMICs). However, knowledge of contextual factors associated with diabetes in LMICs are limited. This study highlights three important lessons on diabetes by identifying and interpreting contextual factors related to its prevention and management within a low-income urban community in Accra, Ghana.

**Methods:**

This was a qualitative study. Data were collected through four focus group discussions (FGDs) with older adults men and women (50+ years) and 18 in-depth interviews with community stakeholders, including traditional leaders, market women, and the Ga Mashie Development Agency. Thematic analysis was conducted to identify key insights on diabetes perceptions, challenges, and cultural practices.

**Results:**

Three key themes emerged from the data: (1) Knowledge does not always translate to action. While participants had extensive knowledge of diabetes risk factors and management, they cited practical constraints that hindered their ability to make behavior changes; (2) Food is more than nutrition. Participants noted that food plays an important role in family, community, and emotional well-being, and (3) Diabetes carries dual meanings. Participants associated diabetes with both individual lifestyle behaviors (e.g., alcohol consumption and sexual activity) and broader environmental exposures (e.g., pollution and chemical contaminants in food).

**Conclusion:**

These results highlight the complexity of diabetes management in an urban poor community context, requiring more than knowledge on diabetes risk factors for behavior change. Addressing personal, communal, and environmental factors, alongside structural barriers, is essential for developing effective, sustainable diabetes management strategies in this setting.

## Introduction

Diabetes is a leading cause of death and disability worldwide, with low- and middle-income countries (LMICs) like Ghana facing a disproportionately high burden (1). In 2019, approximately 463 million people globally were affected by diabetes, and this number is projected to increase to nearly 700 million by 2045 (1, 2). Despite the growing burden of diabetes, research focusing on the contextual and community-specific factors associated with diabetes prevention, management and control remains limited, particularly in LMICs. Understanding community perspectives is crucial for developing effective interventions that are culturally appropriate and align with community residents (3). This approach fosters greater acceptance and sustainability of health interventions (4).

This study is part of the Contextual Awareness, Response, and Evaluation (CARE) Diabetes project, which seeks to describe the contextual factors associated with diabetes in Ga Mashie, a low-income urban community in Accra, Ghana (5, 6). Our team conducted group discussions and individual interviews in Ga Mashie to gather insights into how residents perceive the causes, management, and impacts of diabetes. These discussions covered diverse topics, including history of food systems, community health knowledge, access to healthcare, and broader social and environmental factors affecting health.

In analyzing these conversations, we identified some unanticipated themes, including the disconnect between knowledge and action due to systemic barriers, the cultural and emotional significance of food, and the dual association of diabetes with lifestyle and environmental factors. These insights offer valuable lessons for global diabetes management strategies. While some of these insights are specific to Ga Mashie, others reflect broader issues facing similar urban poor communities. This paper highlights three important lessons on diabetes by identifying and interpreting contextual and cultural themes related to its prevention and management within a low-income urban community in Accra, Ghana. By addressing these contextual and cultural factors, the findings from this study provide actionable insights for designing diabetes management strategies that can be adapted and scaled in similar low-resource urban settings globally.

## Methods

### Data collection

This cross-sectional qualitative study is part of the CARE Diabetes project, that uses epidemiological methods alongside qualitative approaches to generate a contextual understanding of type 2 diabetes (T2D) in an urban poor population (5, 6). Data were collected in a single round through FGDs and in-depth interviews with market women and other key stakeholders involved in food distribution within and outside Ga Mashie, traditional and religious leaders, community influencers, and community members who had insights regarding the history of Ga Mashie. Only participants and researchers were present during data collection. An interview guide (Supplementary material) was used to facilitate discussions, and the guide was pilot tested. No repeat of interviews was conducted. Data collection was conducted from November to December 2022 and details of the interview process have been provided elsewhere (6). Each interview and FGD lasted approximately 45–90 min. Transcripts were not returned to participants for comments or correction. However, a community dissemination event was organized to share the findings with the community. This study received approval from the Ghana Health Service Human Research Ethics Committee (GHS-ERC 017/02/22), the University College London Research Ethics Committee (21541/001), and the Noguchi Memorial Institute for Medical Research Institutional Review Board (NMIMR-IRB CPN 060/21–22 IORG000908).

### Sampling procedure and sample size

**Figure 1:**
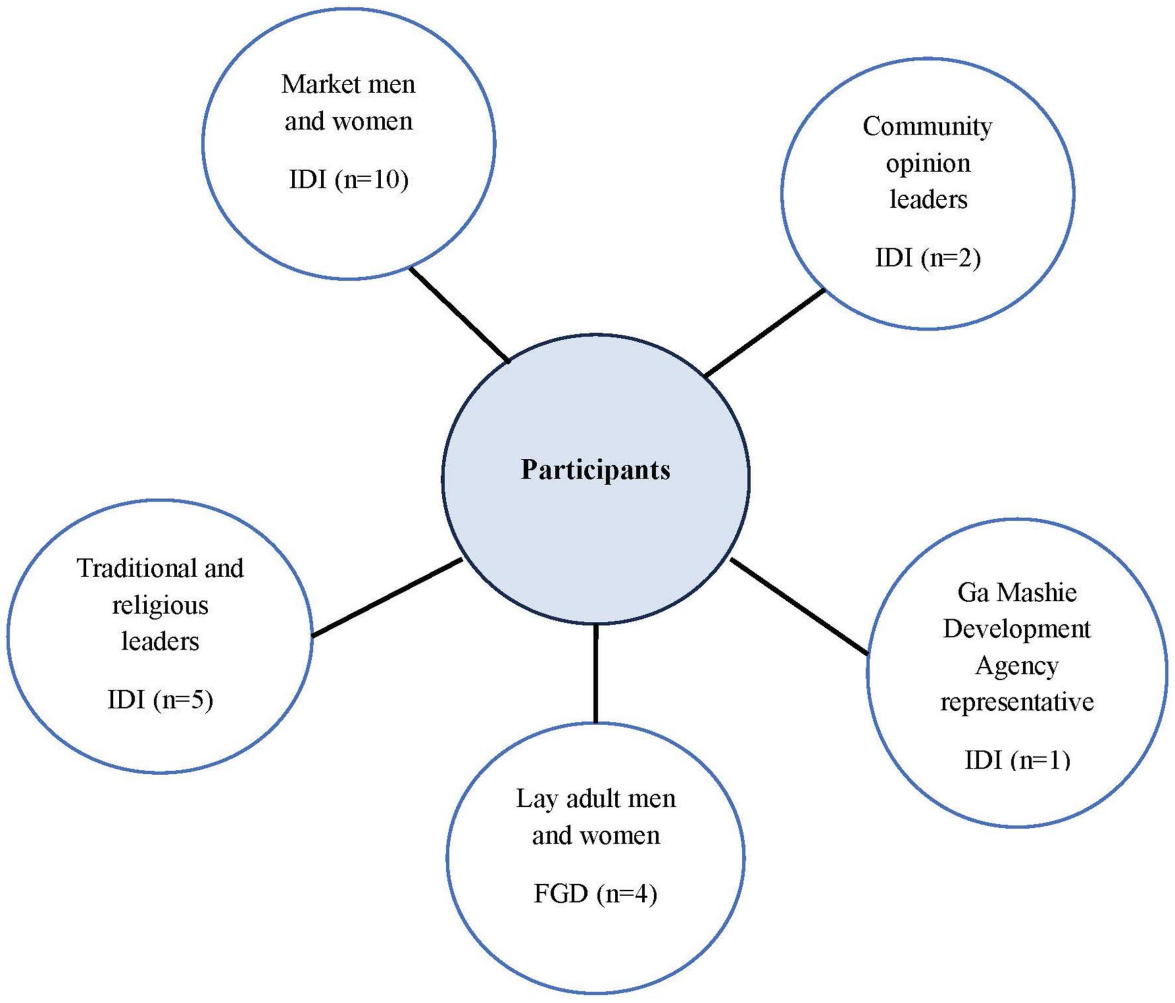
An overview of participants interviewed.

We used a mix of purposive and snowball sampling techniques to recruit key community leaders and members with valuable knowledge on the history of Ga Mashie. A representative from Ga Mashie Development Agency (GAMADA), five traditional and religious leaders, two community opinion leaders and influencers, and 10 market men and women (i.e., people involved in food distribution within and outside the community) were approached to participate in the in-depth interviews (IDIs). Additionally, four focus group discussions (FGDs) were planned with lay adult men and women who were 50 years and above (Figure 1). Two FGDs were conducted with women, and two were conducted with men. Each FGD included 8 participants, totaling 32 individuals (16 men and 16 women). No participant refused to participate or dropped out of the interviews. Participants were purposively sampled based on their diabetes-related knowledge or experiences, and fluency in Ga or Twi. The sample size of 18 IDIs and 4 FGDs was based on achieving thematic saturation, balancing depth and breadth while aligning with similar qualitative studies and capturing diverse community perspectives (7, 8).

### Data analysis

All interviews were audio-recorded and transcribed verbatim by research assistants. All interviews conducted in the local languages (Ga and Twi) were simultaneously translated and transcribed to English. All transcripts were imported into QSR NVivo 11 software to facilitate data coding, analysis, and reporting. Data were analyzed using thematic approach (9). The analysis followed an iterative process, starting with the initial coding of transcripts by three researchers (OAS, LOO and MV) to identify recurring ideas, which were then grouped into broader categories and refined into themes through continuous comparison and team discussions. The coding process started with the identification of deductive codes based on previous qualitative studies, followed by the identification of inductive codes. To ensure inter-coder reliability, the initial coding was cross-checked among the three researchers. Discrepancies were discussed and resolved through consensus. The codes were refined through discussions to capture the main themes and subthemes. Themes emerged from participants’ shared experiences and perceptions, resulting in three main themes with subthemes: (1) knowledge does not always translate to action, (2) food as a cultural and emotional symbol, and (3) dual perceptions of diabetes as both individual and environmental. Each theme was iteratively refined to capture nuanced insights from the participants’ narratives. This qualitative research was reported following the COnsolidated criteria for REporting Qualitative research (COREQ) guidelines (10).

### Reflexivity

Our research team was diverse, consisting of 5 females and 8 males, and included four categories of researchers. All the researchers, except LOO, held a Ph.D. The first category included those with extensive experience in conducting, leading, and publishing qualitative research (OAS, EKD, DS, LB, SBK, RBA, MKK, IAK, DA, and MV). The second category consisted of a researcher with ongoing experience in qualitative research (LOO). The third category included researchers based in Australia (DS), United States (OAS), and United Kingdom (LOO, EF and MV), who also had experience conducting research in Ghana. The fourth category included researchers who were based in academic institutions in Ghana (EKD, LB, SBK, RBA, MKK, SA, and IAK). This diverse composition helped minimize potential biases stemming from academic positions in high-income countries. Additionally, many of the authors were from Ghana. This representation was intentional, as the study was conducted in Ghana and aimed to address inequities often associated with ‘parachute research.’

The interviews were conducted by two trained research assistants, under the supervision of OAS, LOO, EKD and MV. The research assistants received training on the interview guides to familiarize themselves with the questions. We established relationships with participants prior to the study through community engagement activities and preliminary meetings. During these activities, we shared the purpose of the study and the reasons for conducting the research, ensuring that participants understood our motivations and the potential impact of the study on their community.

## Results

### Participant characteristics

A total of 50 participants were interviewed, with a mean age of 54.8 years. Of these, 28 (56.0%) were male, and 22 (44.0%) were female.

### Knowledge does not always equal power

Participants knowledge about diabetes, including its risk factors, prevention, and management strategies mostly align with biomedical theory. However, their ability to translate this knowledge into action was constrained by socioeconomic and environmental factors. Many participants understood the importance of a balanced diet, regular physical activity, and limiting sugar intake to manage or prevent diabetes. Nevertheless, they mentioned that for many of the Ga Mashie population, knowledge about healthy practices is not enough to overcome the systemic barriers they face.

Participants noted economic limitations as a dominant obstacle to diabetes management. Particularly, many of the participants cited the high costs of diabetes medications, blood sugar testing devices, and nutritious foods as significant challenges. One of the participants said “*It’s* (i.e., *diabetes*) *a rich people’s disease, and not for poor people*.” People were well aware of the very serious consequences of poorly managed diabetes: “*it makes you half a human*” said one woman living with the illness. One man was even more blunt: *“If you do not have money you will die.”* The affordability of healthcare and healthy food options was a recurring theme, with community members expressing frustration that, despite understanding what they needed to do to stay healthy, they could not access the necessary resources. Others pointed to a vicious circle in which the people living with diabetes were unable to work, and so unable to afford treatment. In relation to prevention, though there was a clear and quite moralizing perception that people should change their most obviously unhealthy eating and drinking practices, there was also a strong message coming from our respondents that putting dietary knowledge into practice was not easy. *Money, time and space*, were all implicated here.

In addition to economic barriers, participants described living in a context where processed and unhealthy foods are more readily accessible and affordable than traditional, nutrient-dense foods. The shift away from homemade meals to street food and processed snacks reflects broader socioeconomic changes that are difficult for individuals to counteract, even with a solid understanding of healthy eating principles. Participants indicated that time constraints, busy work schedules, and the influence of ‘westernized diets’ were additional barriers to making healthier food choices.

Furthermore, even though participants were aware of the benefits of exercise, limited safe spaces for physical activity posed a challenge. Poor infrastructure, high-density living conditions, and limited recreational areas restricted opportunities for exercise, particularly for women and children. The environmental context, marked by pollution and inadequate urban planning, exacerbated the challenges of maintaining an active lifestyle despite community members’ awareness of its importance to diabetes prevention and management.

### Food is more than nutrition

Participants mentioned that food is far more than just nourishment; it encompasses family, community, enjoyment, and cultural heritage. Older adult participants, in particular, reminisced about the foods of their past, traditional cooking practices, and the flavors created by homemade spices and fermented ingredients. Food, for them, was central to communal identity, contributing to holistic well-being beyond physical health alone. They mentioned that food is about family, community, care, satisfaction and, of course, enjoyment and all of this contributes to health and wellbeing in a wider sense.

They noted that changing economic conditions have shifted dietary practices, as economic pressures have increased dependence on food from street vendors and processed items rather than home-cooked meals. The need for convenience, combined with time and financial limitations, has eroded family meal practices, which many participants noted with nostalgia as symbols of family cohesion and care. Some community members were also anxious about the “hidden” contents in the processed foods they now regularly consumed. This anxiety extended to additives like processed seasoning cubes and other commercial flavorings, which had replaced traditional spices. Concerns also arose around pesticide use in modern agricultural practices, which were believed to impact the quality of vegetables and fruits, leading some to avoid these foods despite their known health benefits.

For many of the participants, food security concerns were exacerbated by fears of contamination and reduced access to nutritious foods, especially as traditional preservation techniques like fermentation and smoking have been partly replaced by refrigeration, a method complicated by intermittent power supply in the community. Overall, participants felt they had lost control over their food environment, surrounded by sugar-laden drinks and processed foods that were affordable but nutritionally compromised, contributing to an unhealthy dietary culture that seemed impossible to avoid.

### Diseases carry multiple meanings

In Ga Mashie, diabetes is interpreted in ways that extend beyond standard biomedical models. Participants in this study sometimes viewed diabetes as “contagious” within their community, though they were aware that it is not infectious in the traditional sense. While most participants understood that diabetes was not contagious, many commented on how it seemed to “spread” through families, emphasizing the communal impact of the disease. For example, one male fisherman said, “*If there’s a family history of the disease, before we know [what] happens the child would develop diabetes*.” This perception sometimes fostered a sense of inevitability, discouraging proactive measures like screenings or lifestyle changes. Others feared stigma and social isolation, refraining from disclosing their condition to avoid being labeled a “carrier.” An opinion leader (male, 77) mentioned that “…*when someone finds out, they may not distance themselves, but they can say something about it that will cause you the person suffering from the disease to be scared.”* Consequently, these beliefs often lead to delayed diagnosis and medical care as well as fatalistic attitudes, undermining the effectiveness of early interventions and efforts to prevent diabetes.

Two broad interpretations of diabetes emerged from the participants. The first, especially prevalent among young men, associated diabetes with lifestyle factors and moral judgments. Participants associated diabetes risk with behaviors they perceived as risky or indulgent, such as alcohol consumption, sexual activity, and involvement in the sports betting economy. Older participants, however, held a second perspective that linked diabetes to environmental and social factors beyond individual control. Many cited crowded living conditions, pollution, and exposure to plastic waste as factors contributing to diabetes. “*People think we are advancing*,” said one man, *“but perhaps we are regressing given the volume of plastics all over.”* Several people remarked that their crowded living conditions did not make it impossible to exercise: “*Our lack of exercise is a result of our own laziness – you can take brisk walks. We do not have designated places for exercise, but we can still manage to do some exercises in our own compounds*.” One woman seemed to be telling us quite clearly not to fetishize exercise but to think instead of everyday movement as beneficial: “*OK as I sit here, assuming I am a little fat, if something is next to you, I should be able to get up and pick it up. That’s also a form of exercise*.”

Participants also expressed concerns about their surrounding food system, including fears of consuming chemically treated or adulterated food items. This sense of environmental vulnerability contrasted with diabetes education’s focus on individual lifestyle modifications. As a result, some community members emphasized a need for broader, “upstream” interventions that could address the external, environmental contributors to diabetes risk, rather than solely focusing on personal choices.

## Discussion

The findings from this study highlight the complexities of addressing diabetes in a socio-economically disadvantaged community like Ga Mashie, where prevention and management efforts are influenced by economic, cultural, and environmental factors. Despite substantial community knowledge about diabetes and its associated risks, the ability to implement this knowledge remains constrained by socio-economic limitations, a situation not uncommon in LMICs. This reinforces the need to look beyond individual behavior change and consider broader contextual challenges that impact health behaviors (3, 6, 11).

Our results align with the work of Moran-Thomas (12), who documented similar experiences in Belize, where diabetes is perceived through both biomedical and social lenses, resulting in culturally embedded understandings of chronic illness. In Ga Mashie, while the community is well-informed about diabetes prevention (6), high inflation, limited income opportunities, and inadequate health resources challenge community residents’ ability to act on that knowledge (5, 6). Financial limitations influence every aspect of diabetes care, from dietary choices to medication adherence, as shown by respondents’ frustration with barriers to accessing care. The gap between knowledge and action is further compounded by the high cost of healthier food options and limited support for managing diabetes, highlighting how economic constraints can lead to health inequities and poor health outcomes in marginalized communities (13, 14). Diabetes prevention efforts should integrate economic support mechanisms, potentially through subsidized medications, affordable monitoring devices, and health insurance schemes tailored to economically disadvantaged groups.

The cultural role of food also emerged as a critical theme, revealing how food is intertwined with social values that affect dietary practices. Traditional Ghanaian food culture emphasizes communal sharing and family bonds, which are both nutritionally and socially enriching (15, 16). However, economic pressures have led to a shift from home-cooked meals to street vendor foods and processed items, which participants noted had led to a decline in traditional eating practices. These dietary shifts not only reduce control over food quality and safety but also disrupt traditional food-sharing practices that promote community cohesion (17). Interventions to improve dietary habits in Ga Mashie and similar communities should focus on culturally tailored solutions, such as promoting locally produced, affordable, and nutritious food options, and not only consider nutrition but also address cultural, economic, and environmental factors that influence food choices. Community-led nutritional programs that re-integrate traditional ingredients and cooking practices could help address the dietary concerns of residents while preserving cultural values (18, 19). Furthermore, respondents expressed anxiety about the nutritional content and safety of processed foods, a concern that reflects the need for public health efforts that go beyond nutritional advice to address structural issues related to food security and food safety.

Our findings also show how chronic diseases like diabetes carry multiple, often conflicting meanings in low-resource settings, where biomedical knowledge coexists with local cultural beliefs and environmental perspectives. While some community members, particularly young men, associated diabetes risk with lifestyle factors and moral judgments about behaviors like alcohol consumption, older residents tended to view diabetes as linked to environmental exposures, such as pollution and chemical contaminants in food. This dual understanding of diabetes, which encompasses both individual behaviors and external environmental risks, suggests that conventional diabetes education focusing only on personal responsibility may be insufficient. Participants’ concerns about living conditions, pollution, and the pervasive presence of processed foods signal a need for “upstream” interventions that address the socio-environmental determinants of health rather than relying solely on individual lifestyle changes (11). Such “upstream” interventions may include food security programs to increase access to nutritious foods (20), urban planning reforms for safe physical activity spaces (21), and policies that target implementation of healthy food environment (22), education initiatives to instill healthy habits in schools (21), and collaboration with local community leaders to ensure that diabetes interventions align with cultural norms and community priorities, to enhance sustainability and impact (23).

This study has a few limitations. The use of purposive and snowball sampling techniques may have introduced selection bias, limiting the generalizability of the results to the wider Ga Mashie community. The sample size, including 18 in-depth interviews and four focus group discussions, might not be fully representative of the diverse perspectives within the community. Finally, the focus on individuals aged 50 and above for the FGDs may have excluded valuable insights from younger generations.

## Conclusion

This study shows the importance of community engagement in understanding and addressing diabetes in low-resource settings. By exploring the perspectives of Ga Mashie residents, we identified key lessons on how diabetes knowledge, cultural values around food, and complex social meanings shape health behaviors. These insights reveal that diabetes interventions need to consider not only individual lifestyle factors but also socio-economic and environmental contexts that affect disease prevention and management. Effective diabetes prevention and management strategies need to integrate culturally relevant approaches, address structural barriers such as food insecurity and limited healthcare access, and involve community members as active partners in the development and implementation of interventions. Future research should focus on scalable, community-driven solutions that consider the broader socio-economic and environmental contexts to ensure sustainable health outcomes.

## Data Availability

The raw data supporting the conclusions of this article will be made available by the authors, without undue reservation.

## References

[1] Lam A Lepe A Wild S Jackson C. Diabetes comorbidities in low-and middle-income countries: an umbrella review. J Glob Health. (2021) 11:11. doi: 10.7189/jogh.11.04040, PMID: 34386215 PMC8325931

[2] International Diabetes Federation. IDF Diabetes Atlas. 9th ed. Brussels, Belgium: International Diabetes Federation (2019).

[3] Hood S Campbell B Baker K. Culturally informed community engagement: Implications for inclusive science and health equity. Research Triangle Park, NC: RTI Press (2023). 37289927

[4] Haldane V Chuah F Srivastava A Singh S Koh G Seng C. Community participation in health services development, implementation, and evaluation: A systematic review of empowerment, health, community, and process outcomes. PLoS One. (2019) 14:e0216112. doi: 10.1371/journal.pone.0216112, PMID: 31075120 PMC6510456

[5] Lule SA Kushitor SB Grijalva-Eternod CS Adjaye-Gbewonyo K Sanuade OA Kushitor MK. The contexual awareness, response and evaluation (CARE) diabetes project: study design for a quantitative survey of diabetes prevalence and non-communicable disease risk in Ga Mashie, Accra, Ghana. Global Health Action (2024) 17:1. doi: 10.1080/16549716.2023.2297513 PMC10851827 38323339

[6] Baatiema L Strachan D Okoibhole L Kretchy I Kushitor M Awuah R. Contextual awareness, response and evaluation (CARE) of diabetes in poor urban communities in Ghana: the CARE diabetes project qualitative study protocol. Glob Health Action. (2024) 17:2364498. doi: 10.1080/16549716.2024.2364498, PMID: 39011874 PMC467110

[7] Boddy C. Sample size for qualitative research. Qual Res J. (2016) 19:426–32. doi: 10.1108/QMR-06-2016-0053

[8] Hennink M Kaiser B Marconi V. Code saturation versus meaning saturation: how many interviews are enough? Qual Health Res. (2017) 27:591–608. doi: 10.1177/1049732316665344, PMID: 27670770 PMC9359070

[9] Attride-Stirling J. Thematic networks: an analytic tool for qualitative research. Qual Res. (2001) 1:385–405. doi: 10.1177/146879410100100307

[10] Tong A Sainsbury P Craig J. Consolidated criteria for reporting qualitative research (COREQ): a 32-item checklist for interviews and focus groups. Int J Qual Health Care. (2007) 19:349–57. doi: 10.1093/intqhc/mzm042, PMID: 17872937

[11] Jack L Jr. Beyond lifestyle interventions in diabetes: a rationale for public and economic policies to intervene on social determinants of health. J Public Health Manag Pract. (2005) 11:357–60. doi: 10.1097/00124784-200507000-00016, PMID: 15958937

[12] Moran-Thomas A. Traveling with Sugar: Chronicles of a global epidemic. 1st ed. University of Chicago Press (2019). doi: 10.2307/j.ctvqr1bjf

[13] Kretchy I Koduah A Ohene-Agyei T Boima V Appiah B. The association between diabetes-related distress and medication adherence in adult patients with type 2 diabetes mellitus: a cross-sectional study. J Diabetes Res. (2020) 2020:1–10. doi: 10.1155/2020/4760624, PMID: 32190697 PMC7071811

[14] Mendenhall E Norris S Shidhaye R Prabhakaran D. Depression and type 2 diabetes in low-and-middle-income countries: a systematic review. Diabetes Res Clin Pract. (2014) 103:276–85. doi: 10.1016/j.diabres.2014.01.001, PMID: 24485858 PMC3982306

[15] Osseo-Asare F. “We eat first with our eyes”, on Ghanaian cuisine. Gastronomica. (2002) 2:49–57. doi: 10.1525/gfc.2002.2.1.49

[16] Roberts J. “Food comes first”: the development of colonial nutritional policy in Ghana, 1900-1950. Glob Food Hist. (2018) 4:168–88. doi: 10.1080/20549547.2018.1465330, PMID: 39989647

[17] Boatemaa S Badasu D de-Graft Aikins A. Food beliefs and practices in urban poor communities in Accra: implications for health interventions. BMC Public Health. (2018) 18:1–12. doi: 10.1186/s12889-018-5336-6, PMID: 29609589 PMC5880073

[18] Batey L DeWitt E Brewer D Cardarelli K Norman-Burgdolf H. Exploring food-based cultural practices to address food insecurity in rural Appalachia. Health Educ Behav. (2023) 50:529–37. doi: 10.1177/10901981231175360, PMID: 37525988 PMC10401894

[19] Metcalfe J McCaffrey J Schumacher M Kownacki C Prescott M. Community-based nutrition education and hands-on cooking intervention increases farmers’ market use and vegetable servings. Public Health Nutr. (2022) 25:2601–13. doi: 10.1017/S1368980022000660, PMID: 35311633 PMC9991668

[20] Gillespie S Poole N van den Bold M Bhavani R Dangour A Shetty P. Leveraging agriculture for nutrition in South Asia: what do we know, and what have we learned? Food Policy. (2019) 82:3–12. doi: 10.1016/j.foodpol.2018.10.012

[21] Bailey R Hillman C Arent S Petitpas A. Physical activity: an underestimated investment in human capital? J Phys Act Health. (2017) 10:289–308. doi: 10.1123/jpah.10.3.289 23620387

[22] Laar A Barnes A Aryeetey R Tandoh A Bash K Mensah K. Implementation of healthy food environment policies to prevent nutrition-related non-communicable diseases in Ghana: national experts’ assessment of government action. Food Policy. (2020) 93:101907. doi: 10.1016/j.foodpol.2020.101907, PMID: 32565610 PMC7299075

[23] Rifkin S. Paradigms lost: toward a new understanding of community participation in health programmes. Acta Trop. (2020) 61:79–92. doi: 10.1016/0001-706x(95)00105-n 8740887

