# Supplementary material for: Three lessons on diabetes for global health professionals, researchers and policy-makers from the people of Ga Mashie
Source: Front Nutr. 2025 Mar 14;12:1534450. doi: 10.3389/fnut.2025.1534450 (PMC11949775; doi:10.3389/fnut.2025.1534450)
Supplement: Supplementary file 1 [file Table_1.DOCX]

**Supplemental File 1. Interview Guide**

**History of health interventions in Ga Mashie**

**Introduction:** – Identity and role of interviewer, general aims of research and role of participant in the process, issues of confidentiality, data access and ownership.

Socio-demographic details to be gathered on standardised form.

1. **General Life History**
   1. Could you tell me a little bit about yourself?

**Prompts:** age, role/status within the community,

1. **History of health intervention**
   1. Can you describe the various health interventions that have been implemented in Ga Mashie that you are aware of? *(Explore current interventions)*
   2. Who are the key initiators/actors/drivers of these health interventions?

***Prompts***: *government, community members, international NGOs, etc)*

- 1. In your opinion, which health interventions have been successful in improving the health of Ga Mashie residents?
  2. Why do you think these interventions were successful?

***Prompts:*** *collaboration with community members, availability of resources, feasibility of intervention, etc.*

- 1. Can you describe the health interventions that you think have been unsuccessful in this community?
  2. Can you please describe the reasons why these interventions were unsuccessful?

***Prompts:*** *limited collaboration with community members in the design and implementation of interventions, limited financial resources/high cost of interventions, non-feasibility of interventions, etc.*

1. **Diabetes intervention**
   1. When you think about diabetes, what interventions have been targeted at prevention, management and control of diabetes within the community?
   2. What interventions have been done towards reducing obesity and overweight in the community?
   3. Can you please describe interventions that have been implemented to improving dietary patterns and habits within the community? *(Explore current interventions)*

***Prompts****: knowledge increase on unhealthy diet*

- 1. In your opinion, which interventions have been successful for improving the prevention, management and control of diabetes within the community?
  2. Why do you think these interventions were successful?

***Prompts:*** *collaboration with community members, availability of resources, feasibility of intervention, etc.*

- 1. Can you describe interventions related to diabetes prevention and management that you think have been unsuccessful in this community?
  2. Can Why do you think these interventions were unsuccessful?
  3. ***Prompts:*** *limited collaboration with community members in the design and implementation of interventions, limited financial resources/high cost of interventions, non-feasibility of interventions, etc.*
  4. In your opinion, what kinds of interventions do you think can help to enhance prevention and management of diabetes in this community?
  5. How do you think these interventions can be implemented within this community? *(Explore actors of implementation)*
  6. To what extent do social-economic activities, political organization and religion shape the prevention and management of diabetes in this community? Take them one after the other and explore how each shapes the prevention and management of diabetes and health care in general.

**Perceptions on changes in food systems and dietary patterns in Ga Mashie**

**Change in food systems**

We would like to ask you some questions about how food and eating have changed in Ga Mashie in your lifetime…….

1. When you think back to your childhood, do you think that the food people eat today is much the same as in the past, or has it changed?
2. If there has been a change, in what ways has this changed?
   1. Are there any foods that people used to eat and no longer eat?
   2. Are there ‘new’ foods that you didn’t see in the past? **Explore here in more detail the composition of a meal.**
3. Are there any changes in WHERE you buy your food from? **Explore what these changes are.**
4. Do you think that people are better or worse fed than in the past? Why?
5. Do you think there have been changes in the way people cook now compared to the past?
   1. If there have been changes, what are these changes? **Explore participant’s perceptions on reasons for the changes.**
   2. If no changes have been observed, why do you think this is so?
6. Do you think there have been changes in how often people cook in this community?
   1. If there have been changes, what are these changes? **Explore participant’s perceptions on reasons for the changes.**
   2. If no changes have been observed, why do you think this is so?
7. In what groups do people eat now and in the past? **Explore groups by gender, and other eating groups**.

***Prompts:*** communal eating

1. When you think about street foods, what is the difference between now and in the past?
   1. What are the changes in street foods composition/other ready-made foods purchased in shops? **Explore reasons for the changes**
   2. What are the changes with frequency of street food consumption or consumption of other ready-made foods purchased in shops? **Explore reasons for the changes**
2. When you think about the taste of foods, do you think this has changed compared to the past?
   1. If yes, what has changed and why?
   2. If no, why has the taste remain the same?
3. Do you think there have been changes with the amount of sugar, salt, additives, and alcohol consumed in this community now compared to the past? **Explore the changes and reason for the changes** **for each of these.**
4. Do you think there are any diseases/medical problems associated with what people eat both now and in the past?
