# Supplementary material for: Three lessons on diabetes for global health professionals, researchers and policy-makers from the people of Ga Mashie
Source: Front Nutr. 2025 Mar 14;12:1534450. doi: 10.3389/fnut.2025.1534450 (PMC11949775; doi:10.3389/fnut.2025.1534450)
Supplement: Supplementary file 2 [file Table_2.pdf]

Supplementary Table 2. COREQ (COnsolidated criteria for REporting Qualitative research) Checklist

Topic	Item No.	Guide Questions/Description	Reported on Page No.
**Domain 1: Research team and reflexivity**			
Personal characteristics			
Interviewer/facilitator	1	Which author/s conducted the interview or focus group?	Reflexivity
Credentials	2	What were the researcher's credentials? E.g. PhD, MD	Reflexivity
Occupation	3	What was their occupation at the time of the study?	Reflexivity
Gender	4	Was the researcher male or female?	Reflexivity
Experience and training	5	What experience or training did the researcher have?	Reflexivity
Relationship with participants			
Relationship established	6	Was a relationship established prior to study commencement?	Reflexivity
Participant knowledge of the interviewer	7	What did the participants know about the researcher? e.g. personal goals, reasons for doing the research	Reflexivity
Interviewer characteristics	8	What characteristics were reported about the interviewer/facilitator? e.g. Bias, assumptions, reasons and interests in the research topic	Reflexivity
**Domain 2: Study design**			
Theoretical framework			
Methodological orientation and Theory	9	What methodological orientation was stated to underpin the study? e.g. grounded theory, discourse analysis, ethnography, phenomenology, content analysis	Data collection
Participant selection			
Sampling	10	How were participants selected? e.g. purposive, convenience, consecutive, snowball	Sampling procedure and sample size
Method of approach	11	How were participants approached? e.g. face-to-face, telephone, mail, email	Sampling procedure and sample size
Sample size	12	How many participants were in the study?	Sampling procedure and sample size
Non-participation	13	How many people refused to participate or dropped out? Reasons?	Sampling procedure and sample size
Setting			
Setting of data collection	14	Where was the data collected? e.g. home, clinic, workplace	Sampling procedure and sample

			size
Presence of non-participants	15	Was anyone else present besides the participants and researchers?	Data collection
Description of sample	16	What are the important characteristics of the sample? e.g. demographic data, date	Participant characteristics
Data collection			
Interview guide	17	Were questions, prompts, guides provided by the authors? Was it pilot tested?	Data collection
Repeat interviews	18	Were repeat interviews carried out? If yes, how many?	Data collection
Audio/visual recording	19	Did the research use audio or visual recording to collect the data?	Data analysis
Field notes	20	Were field notes made during and/or after the interview or focus group?	Data collection
Duration	21	What was the duration of the interviews or focus group?	Data collection
Data saturation	22	Was data saturation discussed?	Sampling procedure and sample size
Transcripts returned	23	Were transcripts returned to participants for comment and/or	Data collection
**Topic**	**Item No.**	**Guide Questions/Description**	**Reported on Page No.**
		correction?	
**Domain 3: analysis and findings**			
Data analysis			
Number of data coders	24	How many data coders coded the data?	Data analysis
Description of the coding tree	25	Did authors provide a description of the coding tree?	Data analysis
Derivation of themes	26	Were themes identified in advance or derived from the data?	Data analysis
Software	27	What software, if applicable, was used to manage the data?	Data analysis
Participant checking	28	Did participants provide feedback on the findings?	Data collection
Reporting			
Quotations presented	29	Were participant quotations presented to illustrate the themes/findings? Was each quotation identified? e.g. participant number	Results
Data and findings consistent	30	Was there consistency between the data presented and the findings?	Results, Discussion
Clarity of major themes	31	Were major themes clearly presented in the findings?	Results
Clarity of minor themes	32	Is there a description of diverse cases or discussion of minor themes?	Results, Discussion

Developed from: Tong A, Sainsbury P, Craig J. Consolidated criteria for reporting qualitative research (COREQ): a 32-item checklist for interviews and focus groups. *International Journal for Quality in Health Care*. 2007. Volume 19, Number 6: pp. 349 – 357
